# A case report of nonsurgical idiopathic normal pressure hydrocephalus differentiated from Alzheimer's dementia: Levetiracetam was effective in symptomatic epilepsy

**DOI:** 10.1002/pcn5.43

**Published:** 2022-09-02

**Authors:** Tetsuro Ishida, Tomonori Murayama, Seiju Kobayashi

**Affiliations:** ^1^ Department of Psychiatry Japan Health Care University Sapporo Japan; ^2^ Department of Psychiatry Asahikawa Keisenkai Hospital Asahikawa Japan; ^3^ Department of Psychiatry Shinyukai Nakae Hospital Sapporo Japan

**Keywords:** case report, dementia, idiopathic normal pressure hydrocephalus, levetiracetam, seizures

## Abstract

**Background:**

Idiopathic normal pressure hydrocephalus (iNPH) is a common form of dementia that causes gait disturbance, cognitive impairment, and urinary incontinence. iNPH is a “treatable dementia” that can be treated with shunt surgery, but this can be ineffective in some cases and can be accompanied by complications. As a result, many patients with iNPH do not undergo surgery. However, there is insufficient evidence on effective treatments other than surgical therapy.

**Case Presentation:**

A 75‐year‐old woman presented to our hospital with a chief complaint of cognitive decline. She showed reduced motivation and inactivity. Brain magnetic resonance imaging showed a high score on the Evans Index (maximum width between bilateral lateral ventricular anterior horns/maximum intracranial cavity in the same slice). The subarachnoid space was enlarged at and below the Sylvian fissure, and narrowed at the higher arcuate region. She was diagnosed with iNPH. However, no shunt surgery was performed; 11 months later, she had a generalized convulsive seizure with loss of consciousness. An electroencephalogram showed generalized epileptic discharges. The possibility of surgery for her iNPH was ruled out. Levetiracetam prevented seizure recurrence and cognitive functions such as spontaneity and motivation were improved.

**Conclusion:**

It is often assumed that surgery is the only effective treatment for patients with iNPH. However, as in the present case, symptomatic epileptic seizures may be a factor in dementia. Even in the absence of surgical treatment, we should examine the cause of dementia in patients with iNPH and consider pharmacological treatment, including antiepileptic drugs.

## Graphical abstract

The disease course and treatment of patients with nonsurgical idiopathic normal pressure hydrocephalus is not clear. However, such patients must be differentiated from other dementias, such as Alzheimer's dementia. They also require medication and support in the psychiatric field.

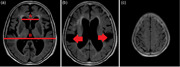

## BACKGROUND

Idiopathic normal pressure hydrocephalus (iNPH) is a condition resulting from cerebrospinal fluid malabsorption disorders, mainly manifesting as gait disturbance, cognitive impairment, and urinary incontinence, in the absence of existing diseases such as subarachnoid hemorrhage or meningitis. iNPH is a common form of dementia in Japan, with a prevalence of 0.2%–3.7% and an estimated annual incidence of approximately 120/100,000 people.^1^, ^2^, ^3^, ^4^, ^5^, ^6^, ^7^ iNPH is a “treatable dementia,” in that it can be effectively treated by shunting. The main shunting techniques are ventriculo‐peritoneal shunts, lumbar‐peritoneal shunts, and ventriculo‐atrial shunts.

In Japan, ventriculo‐peritoneal shunts are performed in 43.2% of cases, lumbar‐peritoneal shunts in 55.1%, and ventriculo‐atrial shunts in 1.7%.^8^ However, shunting is associated with complications such as infection, shunt dysfunction, headache, subdural hygroma, and hematoma. Shunting‐related complications of any kind occur in 18.3% of cases.^8^ Moreover, post‐shunt improvement is highly variable, ranging from approximately (for each time point) 39%–81% at 3–6 months,^9^, ^10^, ^11^ 63%–84% at 1 year,^11^, ^12^, ^13^ 69% at 2 years,^14^ and 60%–74% at 3–6 years^15^ using assessment methods such as the modified Rankin Scale. In addition, some symptoms are more easily improved by surgery than others. Surgery is 60%–77% effective for gait disturbance,^12^, ^16^, ^17^ but only 56%–69% effective for cognitive impairment^14^, ^17^, ^18^ and 52% effective for dysuria.^16^ Furthermore, there is no clear evidence for effective iNPH treatment without surgical therapy, as the disease concept is based on the assumption that shunt surgery improves symptoms. Thus, we report findings concerning the pharmacological treatment of symptomatic epilepsy in a patient with iNPH without surgical treatment, and describe how this improved the patient's symptoms of dementia.

## CASE PRESENTATION

### Chief complaints

A 75‐year‐old woman was admitted to our hospital due to a decline in cognitive function and motivation.

### History of past illness

The patient had become aware of her memory loss 2 years prior and had been diagnosed with Alzheimer's disease at another psychiatric clinic. She was taking donepezil 3 mg/day at the time. She had no physical medical history, including meningitis, subarachnoid hemorrhage, or head trauma.

### Family history

There was no specific history of dementia or hydrocephalus.

### Findings on admission

The patient was 155 cm tall and weighed 53 kg. Her vital signs such as blood pressure and heart rate showed no abnormalities. Psychological examination showed a score of 22/30 on the Hasegawa's Dementia Scale‐Revised (HDS‐R). In the sub‐items of that test, she showed high scores for disorientation and calculation, and low scores for the five‐item recall and verbal fluency, and decreased motivation and spontaneity. Her clinical cognitive functioning was more severe than her HDS‐R score, with a clinical dementia rating (CDR) score of 2.0. The patient had decreased Hamilton Depression Rating Scale‐17 scores in the sub‐items “depressed mood,” “work and activities,” “retardation,” and “hypochondriasis.” However, her total score was 6 points (depression cutoff 7 points).^19^

**Figure 1:**
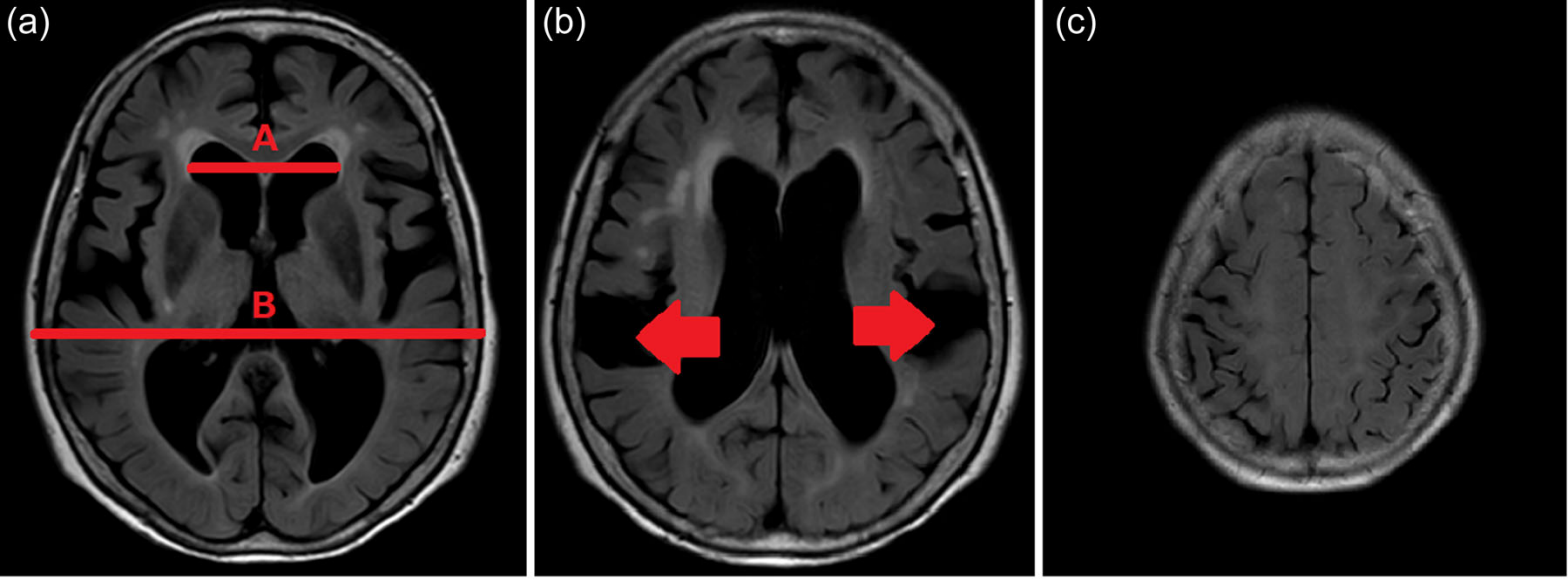
Brain magnetic resonance imaging (MRI). MRI showed enlargement of the ventricles (red lines in A and B) and subarachnoid space below the Sylvian fissure (B, red arrows), and a narrowing at the higher arcuate region (C).

She had no gait or urinary problems, ataxia, or increased or decreased tendon reflexes. The patient's blood tests showed no abnormal thyroid functions nor vitamin deficiencies, and she did not have human immunodeficiency virus or other infections. Brain magnetic resonance imaging (MRI) showed enlargement of both the lateral ventricles and the third and fourth ventricles. Her Evans Index (maximum width between the anterior horns of the bilateral lateral ventricles/largest intracranial cavity in the same slice) was 52/140 mm = 37% (Figure 1a). The subarachnoid space was enlarged at and below the Sylvian fissure (Figure 1b) and narrowed at the higher arcuate region (Figure 1c).

### Diagnosis

On the basis of her cognitive decline and imaging findings, the patient was diagnosed with iNPH.

## OUTCOME AND FOLLOW‐UP

**Figure 2:**
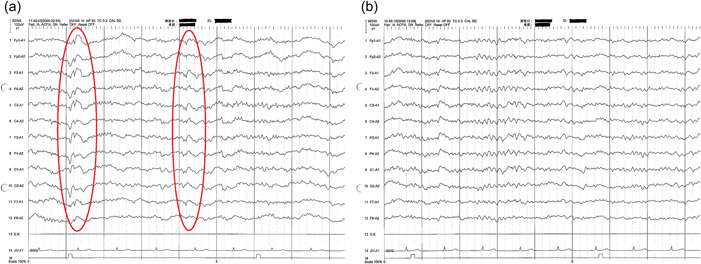
Electroencephalography (EEG). The EEG prior to levetiracetam (LEV) initiation. (A) The spikes and waves (red ovals) and background activity in the θ range. After initiation of LEV, the EEG showed a decrease in spikes and waves, and background activity in the α range (B).

We consulted a neurosurgeon and the patient was administered a tap test. The results showed a cerebrospinal pressure of 65 mmH_2_O (normal 60–150 mmH_2_O) with no fluctuations associated with respiration. Her spinal fluid was colorless and clear and showed no abnormal findings. At 7 days after the tap test, her HDS‐R and CDR showed no improvements in cognitive function. The tap test did not reveal any changes in the patient's cognitive function and her CDR score was unaltered at 2.0. The patient was therefore deemed not to have an indication for iNPH surgery. She then remained in our hospital. Her donepezil dose was increased to 5 mg and goreisan (7.5 mg) was prescribed. However, the patient's cognitive and motor impairments did not improve; 11 months later, she presented with a generalized convulsive seizure with sudden loss of consciousness for 10 min. An intramuscular injection of 10 mg of diazepam caused her seizures to disappear. Another brain MRI showed no change from the previous scan. However, her electroencephalography (EEG) showed background activity in the θ range and extensive spikes and waves, mainly in the bilateral frontal lobe regions (Figure 2a, red ovals). The patient was diagnosed with symptomatic epileptic seizures associated with iNPH. We again consulted with the neurosurgeon. However, as the patient's brain MRI showed no change from the previous one, it was decided that a tap test was not warranted. Instead, intravenous levetiracetam (LEV) 2000 mg/day was started; after 3 days, the LEV route was switched from intravenous to oral.

During 7 days of 2000 mg LEV treatment, the patient had no convulsive seizures with a loss of consciousness, but did have brief impairment seizures one to three times daily, each lasting a few minutes. We decided that LEV was effective for epileptic seizures but the dose was insufficient; we thus increased the dose to 3000 mg. Treatment with 3000 mg LEV resulted in two similar brief impairments in consciousness in the period immediately after the dose increase, but no further seizures. Her EEG showed improved background activity from the θ to α range and decreased spikes and waves (Figure 2b). Three months later, she had the same HDS‐R score as before (22/30). Compared with the previous time, scores decreased in disorientation. However, there was an improvement in motivation and spontaneity. She was now able to converse with other patients and nurses in the hospital dayroom, whereas before she had been dazed, which was accompanied by a CDR score of 1.0. She also gradually became able to perform self‐care activities and is being considered for admission to a nursing home.

## DISCUSSION

### iNPH

#### Clinical symptoms

We will first discuss the clinical presentation of the present case. The main symptoms of iNPH are gait disturbance, dementia, and dysuria. However, the presence of all three is not necessarily required for an iNPH diagnosis. Previous studies worldwide have shown that in iNPH, gait disturbance appears at a frequency of 94%–100%, cognitive impairment at a frequency of 78%–98%, and dysuria at a frequency of 60%–92%^8^, ^13^, ^20^, ^21^, ^22^, ^23^, ^24^; all three symptoms are present in approximately 60% of cases.^21^, ^22^, ^24^ Nevertheless, in a previous study in Japan, the presence of all three symptoms was reported in 12.1% of cases.^8^ In addition, another study in Japan reported that gait disturbance was more severe in patients who consulted a neurosurgeon, and dementia was more severe in patients who consulted a psychiatrist.^25^ This suggests that the symptoms focused on are influenced by the doctor's specialty. The patient described in this paper had dementia, but no gait or urinary disorders. If these symptoms appear in the future, we should consider another tap test and shunt surgery.

#### Imaging

In terms of the cross‐sectional imaging of brain MRI, coronal sections are useful to assess the sulci in the circumflex area,^26^ but horizontal sections can confer the same diagnostic performance as coronal sections if they are imaged to the higher circumflex area.^27^ In iNPH, the Evans Index is usually >0.3. The subarachnoid space is often enlarged in the Sylvian fissure and below, with stenosis in the high arc region.^27^, ^28^, ^29^, ^30^ The presence of these features suggests disproportionately enlarged subarachnoid hydrocephalus (DESH) and can be differentiated with high sensitivity and specificity from atrophy in Alzheimer's disease.^28^, ^29^, ^30^ Our patient was initially diagnosed with Alzheimer's disease, but the diagnosis was changed to iNPH following DESH. The patient's brain MRI met both Evans Index >0.3 and the criteria for DESH. In previous studies, the proportion of iNPH patients who fulfilled both of these criteria was only about 30%.^31^ We should note that when diagnosing iNPH imaging findings alone should not rule out iNPH. Diffusion tensor imaging, single‐photon emission computed tomography, and fluorodeoxyglucose positron emission tomography are also useful in the diagnosis of iNPH. However, a discussion of these techniques is outside the scope of this report.

### Differentiating iNPH from Alzheimer's disease

In the diagnosis of our patient, it was important to differentiate between Alzheimer's dementia and iNPH, but in practice this is often difficult. Indeed, in Europe and Japan, iNPH is considered an independent disease, whereas in the United States, it is classified as a subtype of Alzheimer's disease.^32^

The Evans Index is used to diagnose iNPH; however, this index also fluctuates in other diseases such as Alzheimer's disease. Recent studies have proposed the anteroposterior diameter of the lateral ventricular index (cutoff ratio >0.50) as a new indicator, which is considered useful for discriminating between age‐appropriate atrophy and iNPH.

### Diagnosis according to the Japan Council for Quality Health Care guideline algorithms

The patient was >60 years old and her Evans Index was >0.3. She also had cognitive impairment and DESH but no gait disturbance. Hence, a tap test was performed according to the guideline algorithms.^33^ Because the test showed normal cerebrospinal pressure and no abnormal findings in the cerebrospinal fluid, the patient was diagnosed with probable iNPH. If she has a future shunt procedure and is a shunt responder, a definite iNPH diagnosis can be made.

The algorithm also recommends a repeat tap test or external lumbar drainage (ELD) depending on the tap test results. In ELD, cerebrospinal fluid is eliminated at a rate of 5–10 ml/h or 100–250 ml/day over 1–5 days.^34^ However, ELD requires long‐term catheter placement in the lumbar subarachnoid space, which carries risks of catheter self‐extraction, nerve root pain, and meningitis. Thus, ELD is not regularly performed in Japan. Furthermore, in our region, the COVID‐19 pandemic interfered with nonemergency care—including neurosurgical care—which meant that neither repeat tap tests nor ELD were performed in our patient. This situation highlights the issues around protecting routine medical care during times of high demand; however, this discussion falls outside the scope of our report.

### Epilepsy

Recently, there has been a focus on patients with older‐onset epilepsy, who often present to outpatient dementia clinics.^35^ The relationship between seizures and psychiatric disorders is complex, as they share common factors and interact with each other. In pharmacotherapy, comorbid psychiatric and physical illnesses need to be taken into account when selecting antiepileptic drugs.^36^ Previous studies have shown that postoperative complications of iNPH include seizures, although these are rare, at 0.16%.^37^ However, nonsurgical iNPH presenting with seizures is even rarer, and there has been only one similar case report to our knowledge.^38^ Epilepsy of elderly origin is generally a partial seizure, nonconvulsive status epilepticus (NCSE). The present case was also initially NCSE, and this may have been partly due to the patient's cognitive decline.^39^ However, the patient subsequently presented with convulsive seizures; in view of the presence of iNPH, the patient was diagnosed with symptomatic epilepsy.

### Surgical and drug treatments

As mentioned in the Background, surgical treatment has side effects and complications. If the tap test does not clearly show improved cognitive function, surgery is unnecessary. It is therefore reasonable to assume that surgery was not indicated for the patient. In the absence of surgery, the treatment of patients with iNPH is limited to symptomatic treatment. In our patient, aside from LEV, donepezil and goreisan were prescribed. Donepezil is an antidementia drug that reduces, but does not halt or improve, cognitive decline in dementia patients. Goreisan is a Kampo medicine that regulates water metabolism and is reportedly effective for treating normal pressure hydrocephalus.^40^ Moreover, low‐dose acetazolamide—another diuretic—has been reported to reverse periventricular leukomalacia in iNPH in a small case series.^41^ However, there are no standardized guidelines or large studies supporting the use of goreisan to treat iNPH. An investigation of such supplementary drug therapies for iNPH is therefore needed.

### Treatment outcome

Dementia is usually a progressive disease, therefore, finding that the HDS‐R score of an older patient is the same as 1 year ago is not an indication that the treatment was ineffective, but rather that it prevented cognitive decline. The patient's improvement in the CDR score, which is an index of real‐world quality of life, is also noteworthy. This highlights the importance of adopting multidimensional scales in the assessment of dementia over time.

## AUTHOR CONTRIBUTIONS

Tetsuro Ishida was the patient's primary care physician and contributed to the literature review and manuscript preparation. Tomonori Murayama and Seiju Kobayashi reviewed the literature and contributed to the preparation of the manuscript. All authors gave their approval for the final version to be submitted.

## CONFLICT OF INTEREST

The authors declare no conflict of interest.

## ETHICS APPROVAL STATEMENT

This study was conducted according to the principles of the Declaration of Helsinki.

## PATIENT CONSENT STATEMENT

Informed written consent and a signed release were obtained from the patient for publication of this report and any accompanying images.

## CLINICAL TRIAL REGISTRATION

Not applicable as this is a case report.
